# Envenomation by caterpillars (erucism): proposal for simple pain relief treatment

**DOI:** 10.1186/1678-9199-20-21

**Published:** 2014-05-12

**Authors:** Vidal Haddad, Joel Carlos Lastória

**Affiliations:** 1 Departamento de Dermatologia e Radiologia, Faculdade de Medicina de Botucatu, Universidade Estadual Paulista (UNESP), Caixa Postal 557, Botucatu, SP 18.618-000, Brasil

## To the editor

**Figure 1:**
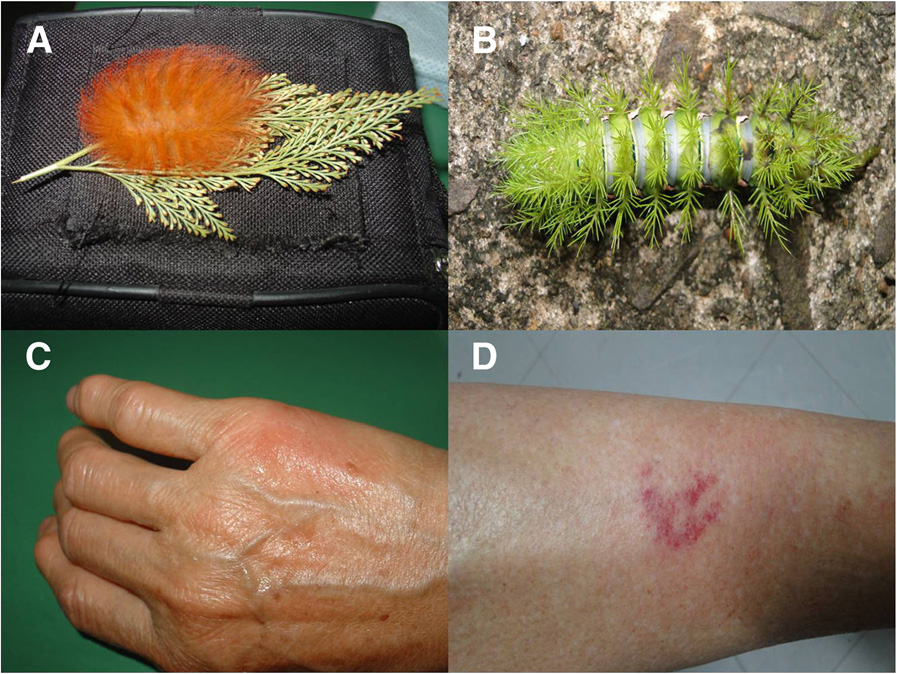
**Caterpillar of the Megalopygidae family (A), Saturniidae family (B) and caterpillar envenomations (C, D) showing mild local inflammation that caused intense pain.** Photos: Vidal Haddad Junior.

Erucism is the name given to injuries caused by moth larvae in humans. The lesions are provoked by caterpillar bristles filled with toxins that penetrate the skin [1-4]. The bristles are hollow and when they enter the skin and break, toxins that contain thermolabile proteins, proteolytic enzymes and histamine are released [2]. The shape of bristles indicates the families of caterpillars mainly associated with injuries: Megalopygidae (fine setae throughout the body) and Saturniidae (setae in small pine tree format, see Figure 1) [3]. The toxins cause immediate severe pain, erythema, edema and immediate lymphangitis (Figure 1). In later stages, there may be vesicles, bullae, erosions, petechiae, superficial skin necrosis, and ulcerations [4]. Some genera (*Lonomia*, *Periga*) can cause severe hemorrhagic syndromes, but most manifestations are limited to excruciating pain and mild to moderate local inflammation [1-4].

Treatment for such injuries is based on cold water compresses and oral analgesics (dipyrone is the rule, but the use of potent analgesics, such as tramadol hydrochloride may be required). When painkillers do not control the pain, nerve anesthetic blockade (lidocaine 4.0 mL for adults) may be employed [1-4]. Based on our past experience, we suggest using serial application of topical commercial anesthetics with 0.25% lidocaine and 0.25% prilocaine in order to greatly decrease or eliminate the pain, these drugs take effect in about half an hour and last for several hours [1,4]. They are easy to apply, does not interfere with the approach adopted for this kind of accident, and it is especially useful for children and patients whose lesion sites do not allow blockades.

## Competing interests

The authors declare that there are no competing interests.
